# Challenges in interpreting cytokine data in COVID-19 affect patient care and management

**DOI:** 10.1371/journal.pbio.3001373

**Published:** 2021-08-06

**Authors:** Stephen Y. Wang, Takehiro Takahashi, Alexander B. Pine, William E. Damsky, Michael Simonov, Yanhua Zhang, Elizabeth Kieras, Christina C. Price, Brett A. King, Mark D. Siegel, Gary V. Desir, Alfred I. Lee, Akiko Iwasaki, Hyung J. Chun

**Affiliations:** 1 Department of Internal Medicine, Yale School of Medicine, New Haven, Connecticut, United States of America; 2 Department of Immunobiology, Yale School of Medicine, New Haven, Connecticut, United States of America; 3 Section of Hematology, Yale School of Medicine, New Haven, Connecticut, United States of America; 4 VA Connecticut Healthcare System, West Haven, Connecticut, United States of America; 5 Department of Dermatology, Yale School of Medicine, New Haven, Connecticut, United States of America; 6 Pfizer, Inc., New York, New York, United States of America; 7 Section of Allergy and Clinical Immunology, Yale School of Medicine, New Haven, Connecticut, United States of America; 8 Section of Pulmonology and Critical Care Medicine, Yale School of Medicine, New Haven, Connecticut, United States of America; 9 Yale Cardiovascular Research Center, Section of Cardiovascular Medicine, Yale School of Medicine, New Haven, Connecticut; 10 Howard Hughes Medical Institute, Chevy Chase, Maryland, United States of America

## Abstract

The importance of cytokine storms in COVID-19 continues to be widely debated. This Perspective article discusses the challenges in using cytokine measurement in COVID-19 and other disease states as we strive to improve our understanding and treatment of COVID-19.

## Teaser

Challenges in using cytokine data are limiting Coronavirus Disease 2019 (COVID-19) patient management and comparison among different disease contexts. We suggest mitigation strategies to improve the accuracy of cytokine data, as we learn from experience gained during the COVID-19 pandemic.

The disease caused by the coronavirus Severe Acute Respiratory Syndrome Coronavirus 2 (SARS-CoV-2) (Coronavirus Disease 2019 (COVID-19)) has resulted in significant morbidity and mortality worldwide. Severe disease is thought to be mediated by a variant of cytokine storm syndrome (CSS), where a hyperinflammatory state characterized by elevated levels of circulating cytokines and chemokines is thought to portend a poor prognosis. The likely significant contribution of CSS in severe COVID-19 infection is supported by demonstrated efficacies of broad immunomodulatory agents, including corticosteroids and Janus kinase (JAK) inhibitors, in improving clinical outcomes in hospitalized patients with COVID-19 [1–3]. While multiple studies have described elevated levels of pro-inflammatory cytokines in severely ill patients hospitalized with COVID-19 [4], a subset of studies have questioned whether CSS is indeed an important mechanistic driver of COVID-19 based on the observation that cytokine elevation in COVID-19 may be more muted compared to conditions such as acute respiratory distress syndrome (ARDS), sepsis, and cytokine release syndrome related to chimeric antigen receptor T-cell therapy [5,6].

The lack of a gold standard for measuring cytokines using multiplexed approaches and significant variation in measurements among different assays raises the concern that the discrepancies among studies in COVID-19 and other diseases may be related to differences in the laboratory vendors or specific assays used for measurement. We experienced this discrepancy in cytokine measurements firsthand in our health system, where cytokine panels were routinely ordered for many of over 4,000 patients hospitalized with COVID-19, and a quantitative multiplex bead assay for a panel of 12 cytokines was conducted by an off-site Clinical Laboratory Improvement Amendments (CLIA)-accredited vendor (Lab A). Peak cytokine levels from 1,328 patients, from whom serial cytokine levels were obtained throughout their hospitalization, were extracted from the electronic medical records and the recently established Yale DOM-CovX database [7] (**Table 1**). In parallel, plasma was collected from a subset of 247 patients hospitalized with COVID-19 and were enrolled in research studies, and multiplex cytokine levels were measured by a secondary laboratory (Lab B), which used a different type of multiplex assay, and these patients were categorized into intensive care unit (ICU) and non-ICU, based on their hospitalization status at the time of sample collection [7,8]. While many of the cytokines measured by Lab B were found to be elevated and correlated with disease severity and outcomes including mortality [7], the cytokine values from the CLIA-certified Lab A reported almost all of the cytokines measured being below level of detection (defined as concentration <5 pg/mL), including from those who were critically ill (**Table 1**). Given this high degree of discrepancy, we utilized a third vendor (designated Lab C, a CLIA-certified lab that utilizes Meso Scale Discovery Electrochemiluminescence (MSD-ECL) platform) to conduct confirmatory analyses on interleukin (IL)-6, IL-8, interferon gamma (IFN-γ), and tumor necrosis factor alpha (TNF-α) and found a strong correlation with Lab B for IL-6, IL-8, IFN-γ, but not for TNF-α (**Table 1**). Such variability in the cytokine levels, including large differences even between 2 CLIA-certified laboratories, highlights the challenges of interpreting the data in a meaningful way to inform patient care and management.

**Table 1:** Median values of cytokine concentrations among hospitalized patients with COVID-19 by severity of illness.

Cytokine	Lab A (pg/mL (IQR))	Lab B (pg/mL (IQR))	Lab C (pg/mL (IQR))
**ICU**			
	*n =* 408	*n =* 95	*n* = 25
IL-6	57.8 (9.0–279.5)	259.8 (85.4–819.3)	113.4 (20.5–381.9)
IL-8	<5.0	23.86 (12.5–54.8)	21.4 (11.9–41.0)
IFN-γ	<5.0	12.1 (7.2–35.2)	13.4 (5.4–21.2)
TNF-α	<5.0	106.6 (52.5–173.2)	4.9 (2.9–6.9)
IL-10	14.7 (7.0–25.8)	17.0 (6.1–37.8)	
IL-1B	<5.0	10.4 (5.8–25.5)	
IL-2	<5.0	0.9 (0.3–2.0)	
IL-4	<5.0	0.1 (0.0–1.6)	
IL-5	<5.0	9.8 (4.4–20.6)	
IL-12	<5.0	69.24 (37.0–149.0)	
IL-13	<5.0	61.2 (30.5–183.6)	
IL-17	<5.0	4.4 (1.7–8.8)	
**Non-ICU**			
	*n* = 920	*n* = 152	
IL-6	8.0 (5.0–28.75)	15.6 (5.1–43.6)	
IL-8	<5.0	14.8 (5.0–30.2)	
IFN-γ	<5.0	9.5 (4.5–21.0)	
TNF-α	<5.0	75.74 (50.5–111.4)	
IL-10	9.0 (5.0–16.0)	7.4 (2.1–19.7)	
IL-1B	<5.0	7.6 (3.3–16.2)	
IL-2	<5.0	0.2 (0.0–0.8)	
IL-4	<5.0	0.0 (0.0–1.0)	
IL-5	<5.0	6.3 (3.8–11.9)	
IL-12	<5.0	80.0 (43.0–160.7)	
IL-13	<5.0	40.4 (24.8–79.3)	
IL-17	<5.0	2.3 (0.4–6.3)	

COVID-19, Coronavirus Disease 2019; ICU, intensive care unit; IFN-γ, interferon gamma; IL, interleukin; TNF-α, tumor necrosis factor alpha.

We sought to further place our findings in the context of cytokine concentrations reported by other groups. While a number of initial reports demonstrated marked elevation in cytokine levels in hospitalized patients with COVID-19 including strong correlation of IL-6 and IL-8 levels with disease severity [9], recent reports [5,10] observed that cytokine values in COVID-19 were lower than those reported from other inflammatory syndromes. Importantly, while we recognize the challenges of comparing cytokine concentrations between different vendors, the concentrations of IL-6 and IL-8 in our critically ill ICU cohort, as measured by Lab B, were actually higher than the values reported in other inflammatory states including those with ARDS [5].

The wide discrepancies in the reported cytokine levels continue to generate questions regarding the importance of CSS in COVID-19. These questions not only center around cytokines thought to be playing a central role in COVID-19, including IL-6 and IL-8, but also multiple other cytokines that were elevated in severe COVID-19 patients from one laboratory vendor but not another in our own experience (**Table 1**). Such discrepancies in the cytokine levels not only have critical implications in our understanding of the pathobiology of disease, but also likely influence choice of therapeutics.

While the natural course of disease, treatments administered, and other factors can account for some aspects of the variability in cytokine concentrations, it is apparent that additional non-disease–related factors related to laboratory accuracy may contribute to the differences observed among different vendors and platforms. Assays undoubtedly have variations in precision; however, there may also be differences in results within each laboratory due to different antibodies, calibration standards, detection reagents, and methods used for the assays [11]. Knight and colleagues recently studied laboratory proficiency testing data between 2015 and 2018 using samples sent to various United States laboratories. Their study found statistically significant variation in values by the type of assay used for IL-6, IL-8, and TNF-α, which were the most implicated cytokines in the pathophysiology of COVID-19. Furthermore, the authors found enormous variability in cytokine values even within the same laboratories and concluded that given significant variability, standardized testing is needed for cytokines [11].

While cytokine analyses of patients with COVID-19 have the potential to advance our understanding of the disease and have been used in many COVID-19 studies, given the key limitations highlighted here, comparison of cytokine data from different disease states using nonuniform assay platforms are indeed like comparing “apples and oranges.” Such generalized comparison of COVID-19 populations with historical controls of ARDS, sepsis, and other disease states lacks the necessary fidelity to reach meaningful conclusions. While the race to achieve a greater understanding and meaningful therapeutic impact during the height of the pandemic led many clinicians and scientists to rush to identify diagnostic tests and treatment modalities, increased vigilance as we transition to the next phase of COVID-19 will be critical to achieve deeper understanding of the pathobiology that will ultimately yield better therapies and clinical outcomes.

Hence, when evaluating cytokine data, we recommend the following: (1) rigorous validation of the assays to ensure accuracy of the data output; (2) avoidance of comparing data from different assay platforms when comparing different disease states; and (3) evaluating the data in the context of disease mechanisms and therapeutic efficacy. With respect to the third point, thus far in the course of the pandemic, broader immunosuppressive therapies such as corticosteroids and JAK inhibitors have yield far more efficacious responses [1–3] than cytokine specific therapeutics such as anakinra and canakinumab (IL-1) [12], mavrilimumab (granulocyte-macrophage colony-stimulating factor (GM-CSF)) [13], and even sarilumab and tocilizumab (IL-6), with demonstrated efficacy in some, but not other, randomized clinical trials [14]. Results from these clinical trials, along with mechanistic studies demonstrating a broader inflammatory state, suggest that CSS may indeed play an important role in COVID-19 pathogenesis [7]. Nevertheless, the scientific community needs to proceed with caution when interpreting cytokine data, especially given the importance of cytokine modulation to combat COVID-19, a disease that is far from being eradicated.

## Abbreviations

ARDS: acute respiratory distress syndrome
CLIA: Clinical Laboratory Improvement Amendments
COVID-19: Coronavirus Disease 2019
CSS: cytokine storm syndrome
GM-CSF: granulocyte macrophage colony-stimulating factor
IFN-γ: interferon gamma
IL: interleukin
JAK: Janus kinase
MSD-ECL: Meso Scale Discovery Electrochemiluminescence
SARS-CoV-2: Severe Acute Respiratory Syndrome Coronavirus 2
TNF-α: tumor necrosis factor alpha

## References

[1] Guimarães PO, Quirk D, Furtado RH, Maia LN, Saraiva JF, Antunes MO, et al. Tofacitinib in Patients Hospitalized with Covid-19 Pneumonia. N Engl J Med. 2021. doi: 10.1056/NEJMoa2101643 34133856 PMC8220898

[2] Kalil AC, Patterson TF, Mehta AK, Tomashek KM, Wolfe CR, Ghazaryan V, et al. Baricitinib plus Remdesivir for Hospitalized Adults with Covid-19. N Engl J Med. 2021;384(9):795–807. doi: 10.1056/NEJMoa2031994 33306283 PMC7745180

[3] RECOVERY Collaborative Group, Horby P, Lim WS, Emberson JR, Mafham M, Bell JL, et al. Dexamethasone in Hospitalized Patients with Covid-19. N Engl J Med. 2021;384(8):693–704. doi: 10.1056/NEJMoa2021436 32678530 PMC7383595

[4] Huang C, Wang Y, Li X, Ren L, Zhao J, Hu Y, et al. Clinical features of patients infected with 2019 novel coronavirus in Wuhan, China. Lancet. 2020;395(10223):497–506. doi: 10.1016/S0140-6736(20)30183-5 31986264 PMC7159299

[5] Leisman DE, Ronner L, Pinotti R, Taylor MD, Sinha P, Calfee CS, et al. Cytokine elevation in severe and critical COVID-19: a rapid systematic review, meta-analysis, and comparison with other inflammatory syndromes. Lancet Respir Med. 2020;8(12):1233–44. doi: 10.1016/S2213-2600(20)30404-5 33075298 PMC7567529

[6] Sinha P, Matthay MA, Calfee CS. Is a “Cytokine Storm” Relevant to COVID-19? JAMA Intern Med. 2020;180(9):1152–4. doi: 10.1001/jamainternmed.2020.3313 32602883

[7] Meizlish ML, Pine AB, Bishai JD, Goshua G, Nadelmann ER, Simonov M, et al. A neutrophil activation signature predicts critical illness and mortality in COVID-19. Blood Adv. 2021;5(5):1164–77. doi: 10.1182/bloodadvances.2020003568 33635335 PMC7908851

[8] Lucas C, Wong P, Klein J, Castro TB, Silva J, Sundaram M, et al. Longitudinal analyses reveal immunological misfiring in severe COVID-19. Nature. 2020;584(7821):463–9. doi: 10.1038/s41586-020-2588-y 32717743 PMC7477538

[9] Del Valle DM, Kim-Schulze S, Huang HH, Beckmann ND, Nirenberg S, Wang B, et al. An inflammatory cytokine signature predicts COVID-19 severity and survival. Nat Med. 2020;26(10):1636–43. doi: 10.1038/s41591-020-1051-9 32839624 PMC7869028

[10] Mudd PA, Crawford JC, Turner JS, Souquette A, Reynolds D, Bender D, et al. Distinct inflammatory profiles distinguish COVID-19 from influenza with limited contributions from cytokine storm. Sci Adv. 2020;6(50). doi: 10.1126/sciadv.abe3024 33187979 PMC7725462

[11] Knight V, Long T, Meng QH, Linden MA, Rhoads DD. Variability in the Laboratory Measurement of Cytokines. Arch Pathol Lab Med. 2020;144(10):1230–3. doi: 10.5858/arpa.2019-0519-CP 32401053

[12] The CORIMUNO-19 Collaborative group. Effect of anakinra versus usual care in adults in hospital with COVID-19 and mild-to-moderate pneumonia (CORIMUNO-ANA-1): a randomised controlled trial. Lancet Respir Med. 2021;9(3):295–304. doi: 10.1016/S2213-2600(20)30556-7 33493450 PMC7825875

[13] Cremer PC, Abbate A, Hudock K, McWilliams C, Mehta J, Chang SY, et al. Mavrilimumab in patients with severe COVID-19 pneumonia and systemic hyperinflammation (MASH-COVID): an investigator initiated, multicentre, double-blind, randomised, placebo-controlled trial. Lancet Rheumatol. 2021;3(6):e410–e8. doi: 10.1016/S2665-9913(21)00070-9 33754144 PMC7969143

[14] Rubin EJ, Longo DL, Baden LR. Interleukin-6 Receptor Inhibition in Covid-19—Cooling the Inflammatory Soup. N Engl J Med. 2021;384(16):1564–5. doi: 10.1056/NEJMe2103108 33631064 PMC7944949

